# London County Council Tuberculosis Scheme: The Position of the Hospitals

**Published:** 1914-05-30

**Authors:** 


### LONDON COUNTY COUNCIL TUBERCULOSIS SCHEME.

### The Position of the Hospitals.

It is now fifteen months since the London County Council instructed its Public Health Committee to prepare a scheme for dealing with tuberculosis throughout the administrative county. The Public Health Committee have been anxious to encourage the provision of a complete network of dispensaries, being impressed with the potential value of these institutions as a means of discovering contact cases at an early stage and as a necessary first unit in any complete scheme. Their object throughout has been to make the fullest possible use of the many agencies already available to ensure the co-operation of the great London hospitals. The efforts of the Council, in their opinion, may most usefully be directed to linking up all these agencies and others still to be created, so that the progress of a patient may be supervised from the time he first develops the disease. The Council's funds should be utilised to encourage the provision of fresh accommodation or to secure the keeping open of accommodation which is in financial danger. Special attention should also be devoted to the provision of residential accommodation for tuberculous children, as the Committee are confident that it is in this direction that expenditure will be most profitably incurred. It has been suggested that the County Council itself might be called upon to own and manage institutions, but the Committee are of opinion that adequate provision can be made under arrangements with the Metropolitan Asylums Board and the authorities of hospitals and sanatoria. For the present the scheme must be largely provisional owing to the lack of definite figures as to the amount of accommodation which may ultimately be required. It has been thought advisable, especially in the case of adults, to wait until the Council has had experience of the numbers of cases recommended for treatment before binding the Council to make use of extensive accommodation for prolonged periods.

### The Advisory Board.

Under this scheme dispensary treatment will be provided by the local sanitary authorities, and towards this treatment the County Council is prepared to contribute, so far as uninsured persons are concerned, grants not exceeding 50 per cent. of the cost which would otherwise fall on the funds of the local authorities. These grants will be made subject to certain conditions, one of which is that the dispensary must be linked up with a hospital. Arrangements for residential treatment, both in hospitals and sanatoria, will be made by the Council so far as uninsured persons are concerned, and by the London Insurance Committee for insured persons. An advisory board, consisting of representatives of hospitals, dispensaries, and sanatoria, will be set up to assist in securing uniformity in the selection of patients. This board should also be of great assistance in advising the Council on the most useful type of co-operation. Recommendations for the provision of residential treatment for a tuberculous person will be received from local medical officers of health, who will forward a report by the physician who has been in attendance on the patient, either in his home or at the dispensary or hospital, as to the clinical aspects of the case, and their own reports as to the home conditions of the patient. When these reports indicate the suitability of residential treatment, arrangements will be made for admission. If, however, there appears to be any doubt as to the suitability of the case or as to the relative degree of urgency of a number of cases when a demand for beds exceeds the immediate supply, the cases will be submitted to a rota of physicians to be selected by the advisory board. When a patient leaves the residential institution arrangements will be made for the local dispensary or other agency to continue his treatment. A condition of receiving residential treatment under the scheme is that the patient must undertake to remain under medical supervision on discharge. The proposals for an advisory board consisting of representatives of all the participating institutions, and for a rota to examine cases on behalf of the Council, were discussed at a conference with representatives of the principal London hospitals last week, when a resolution was passed, by a large majority, in which the representatives expressed their general approval, and intention to advise the hospitals with which they are connected to appoint members on the advisory board.

### The Amount of Residential Accommodation Needed.

The Public Health Committee have experienced considerable difficulty in preparing an estimate of the amount of residential accommodation which will be required during the current financial year. Provision in respect of insured persons, most of whom are males, is being made by the London Insurance Committee, and it would therefore appear that the adults for whom the Council will have to provide will, for the greater part, be women. It is from this circumstance that difficulty has principally arisen, as it has been found almost impossible to frame a reliable estimate of the number of uninsured tuberculous women likely to require residential treatment. So far as the children are concerned the difficulty has been somewhat less owing to the knowledge which the Council has of the health of children of school age. The Committee are of opinion that by the end of the current financial year 160 beds will be required in hospitals and 240 in sanatoria for adults, while as an initial provision 150 beds should be obtained in residential institutions for children. As to the Council's ultimate liability no reliable estimate can be formed until after experience of the scheme's working.

In endeavouring to secure residential accommodation the Public Health Committee have proceeded on the principle that for the present year the Council's funds as far as possible should be utilised to make existing accommodation available. So far as hospital beds are concerned, it appears that there is almost sufficient accommodation at reasonable prices for immediate needs, but this is not the case with sanatorium beds, either for adults or children. The Committee, therefore, consider that the Council should encourage existing suitable institutions to increase their accommodation. The London Insurance Committee have succeeded in obtaining the hospital and sanatorium accommodation they require at a price not exceeding 30s. a week for each bed, and the Public Health Committee have negotiated on the same terms.

It is proposed that in the first instance agreement should be entered into for a period expiring on or about June 30, 1915, but there is little doubt that some, if not all, will have to be renewed for a further period. To meet the conditions laid down by most of the institutional authorities it is proposed that beds shall be taken as required, and that when a bed has been once taken it shall be retained for the remainder of the contract period. Subject in some cases to the adjustment of certain questions still under discussion beds can be secured on these conditions at the following institutions: Brompton, City of London (Victoria Park), King's College, Metropolitan, Royal Chest (City Road), Royal Free, St. Bartholomew's, St. Mary's, University College, and Westminster Hospitals, and at the Fairlight (Hastings), Frimley (in connection with Brompton Hospital), Whitmead (Surrey), and Maltings Farm (Suffolk) Sanatoria. It is also understood that the authorities of King's College Hospital are prepared to place a new ward containing 24 beds, when ready, at the Council's disposal for the sum of £1,800 a year, and it is proposed to accept the offer. This accommodation, however, will probably not be available until towards the close of the year. At present the sanatorium accommodation available is considerably below the Council's requirements, but it is hoped that some additional beds can be obtained at the rate of 30s. a week from certain institutions at which the price at present asked is in excess of that amount. Even with these additions the Metropolitan Asylums Board have been asked whether 100 beds can be placed at the Council's disposal for use during the current year.

No account has been taken in these calculations of advanced and chronic cases. It appears that there are at present in Poor-Law institutions in London about 2,300 tuberculous persons, most of whom come within this category, and of whom about 86 per cent. are uninsured persons. The Public Health Committee think that the question of providing for this class should be dealt with on behalf of the Council by the Metropolitan Asylums Board, who might possibly arrange to take over some of the infirmaries with this end in view.

With regard to residential accommodation for children, the Committee are of opinion that for ordinary cases the weekly charge for each bed should not exceed 20s. The East Anglian Sanatorium at Nayland and the Victoria Home, Margate, are prepared to receive children sent by the Council, and the Asylums Board has been asked whether it can provide accommodation for children at its institution at Carshalton. It is also proposed to enter into negotiations with the authorities of the Alexandra Hospital, who are thinking of building a children's convalescent home near London. The cost of the scheme for the year 1914-15 is estimated at £28,921, of which £8,928 will be required for hospital beds, £13,439 for sanatorium beds, £5,454 for children's beds, and £1,100 for travelling and incidental expenses. On this estimate the Government contribution would amount to £14,250. The cost for 1915-16 is put at roughly £40,000, towards which the Government would contribute £20,000.

### The Discussion.

The scheme came before the London County Council at its meeting on Tuesday, and was approved after some criticism as to details. Mr. J. A. Dawes, M.P., chairman of the London Insurance Committee, said that from his point of view there was one very great blot on the whole scheme—that it dealt with uninsured persons only. Other counties and big boroughs had taken the heroic view and had included in their scheme the treatment of the whole of the tuberculous population. That was the only way they could ever get a comprehensive and entirely satisfactory scheme. He was afraid the Committee had very much underestimated the number of beds they would require both in sanatoria and hospitals. They estimated 240 beds for adults and 160 for children, but the Insurance Committee had something over 700 persons in institutions, and insured persons were only about one-third of the population.

Mr. Oscar Warburg, chairman of the Public Health Committee, said that the provision which was suggested in the scheme was in no sense an indication of the amount which would be ultimately required. It was quite uncertain to what extent women patients would be prepared to leave their homes and take advantage of residential treatment. The hospitals were anxious to co-operate with the Council in every possible manner.

